# Maternal education and its association with maternal and neonatal adverse outcomes in live births conceived using medically assisted reproduction (MAR)

**DOI:** 10.1186/s40748-023-00170-4

**Published:** 2023-12-01

**Authors:** Cassie L. Hobbs, Christina Raker, Gabrielle Jude, Jennifer L. Eaton, Stephen Wagner

**Affiliations:** 1 Department of Obstetrics and Gynecology, Women and Infants Hospital, Warren Alpert Medical School of Brown University, Providence, RI USA; 2 Department of Obstetrics and Gynecology, Division of Research, Women and Infants Hospital, Warren Alpert Medical School of Brown University, Providence, RI USA; 3 Division of Reproductive Endocrinology and Infertility, Department of Obstetrics and Gynecology, Women and Infants Hospital, Warren Alpert Medical School of Brown University, Providence, RI USA

**Keywords:** Maternal education, Medically assisted reproduction, Maternal adverse outcomes

## Abstract

**Background:**

To examine the association between maternal education and adverse maternal and neonatal outcomes in women who conceived using medically assisted reproduction, which included fertility medications, intrauterine insemination, or in vitro fertilization.

**Methods:**

We conducted a retrospective cohort study utilizing the US Vital Statistics data set on national birth certificates from 2016 to 2020. Women with live, non-anomalous singletons who conceived using MAR and had education status of the birthing female partner recorded were included. Patients were stratified into two groups: bachelor’s degree or higher, or less than a bachelor’s degree. The primary outcome was a composite of maternal adverse outcomes: intensive care unit (ICU) admission, uterine rupture, unplanned hysterectomy, or blood transfusion. The secondary outcome was a composite of neonatal adverse outcomes: neonatal ICU admission, ventilator support, or seizure. Multivariable modified Poisson regression models with robust error variance adjusted for maternal age, race, marital status, prenatal care, smoking during pregnancy, neonatal sex, and birth year estimated the relative risk (RR) of outcomes with a 95% confidence interval (CI).

**Results:**

190,444 patients met the inclusion criteria: 142,943 had a bachelor’s degree or higher and 47,501 were without a bachelor’s degree. Composite maternal adverse outcomes were similar among patients with a bachelor’s degree (10.1 per 1,000 live births) and those without a bachelor’s degree (9.4 per 1,000 live births); ARR 1.05, 95% CI (0.94–1.17). However, composite adverse neonatal outcomes were significantly lower in women with a bachelor’s degree or higher (94.1 per 1,000 live births) compared to women without a bachelor’s degree (105.9 per 1,000 live births); ARR 0.91, 95% CI (0.88–0.94).

**Conclusions:**

Our study demonstrated that lower maternal education level was not associated with maternal adverse outcomes in patients who conceived using MAR but was associated with increased rates of neonatal adverse outcomes. As access to infertility care increases, patients who conceive with MAR may be counseled that education level is not associated with maternal morbidity. Further research into the association between maternal education level and neonatal morbidity is indicated.

## Introduction

In the United States, the use of medically assisted reproduction (MAR) has increased steadily over the past two decades, with current reports from the Centers for Disease Control and Prevention (CDC) citing 12 in every 100 patients aged 15–49 have used infertility services [1, 2]. The National Vital Statistics System data dictionary defines MAR as the use of fertility-enhancing drugs, artificial insemination, intrauterine insemination or the use of assisted reproductive technology [e.g., in vitro fertilization (IVF), or gamete intrafallopian transfer (GIFT)]. Despite the widespread use of medically assisted reproduction, many studies have raised concern regarding the adverse obstetrical and perinatal outcomes associated with pregnancies conceived using infertility treatments, though some of these risks may be intrinsic to the infertile population itself [3–7]. Continued investigation into the etiology of the increased risk of morbidity and interventions to optimize preconception health and counseling is necessary to minimize maternal and neonatal complications associated with MAR [5].

In recent years, there has been increased attention drawn to identifying causal links of social variables and their determinative role in obstetric morbidity, one factor being the level of maternal education [8]. There is considerable evidence that shows low level of maternal education is a predictor of poor perinatal outcomes including low Apgar score, preterm birth, neonatal low birth weight, and mortality [9–11]. Further, many population-based studies have shown an association between low maternal education and increased risk of poor maternal outcomes, namely post-cesarean complications, sepsis, ICU admissions, eclampsia, and death [12–14]. However, limited literature exists that describes the impact of maternal education on maternal-fetal morbidity in patients who conceive using MAR. Further research into maternal education as a predictor of adverse obstetrical outcomes in patients who use MAR may influence counseling and delivery of fertility interventions and further inform frameworks for addressing structural determinants of health in pregnancy.

In this present study, we conducted a retrospective national cohort study using the US National Birth Certificate dataset from 2016 to 2020 with the primary objective of examining the association between maternal education level and adverse maternal and neonatal outcomes in live births conceived using MAR. We hypothesized that higher maternal education levels would be associated with lower rates of both maternal and neonatal morbidity.

## Methods

This was a population-based retrospective cohort study using US National Vital Statistics on linked birth and infant death data from 2016 to 2020. The study population included live births from women who conceived using MAR. The US National Vital Statistics defines medically assisted reproduction as the use of fertility-enhancing drugs, artificial insemination or intrauterine insemination, and assisted reproductive technology including in vitro fertilization (IVF) and gamete intrafallopian transfer (GIFT). The exclusion criteria were: non-US resident, birth outside a hospital, non-singleton gestation at time of delivery, gestational age less than 24 weeks or greater than 41 weeks, diabetes mellitus or gestational diabetes, gestational or chronic hypertension, preeclampsia, known fetal anomaly, spontaneous or unknown conception or unknown education level. Deliveries with missing outcome data were excluded from the analysis.

The exposure variable for this study was level of maternal education of the birthing partner: specifically, having less than a bachelor’s degree or having a bachelor’s degree or higher. The primary outcome was a composite of maternal adverse outcomes including: intensive care unit (ICU) admission, uterine rupture, unplanned hysterectomy, or need for a blood transfusion. The secondary outcome was a composite of neonatal adverse outcomes including: neonatal ICU admission, ventilator support, or seizure. We elected to utilize these variables to form the composite adverse outcome and exclude individuals with hypertensive disorders and pregestational or gestational diabetes to maintain consistency with previously published literature [15, 16]. A preplanned subset analysis stratified maternal morbidity by MAR technique. Multivariable modified Poisson regression models with robust error variance adjusted for maternal age, race, marital status, prenatal care, smoking during pregnancy, neonatal sex, and birth year estimated the relative risk (RR) of outcomes with a 95% confidence interval (CI). If mothers or neonates suffered more than one adverse outcome they were counted only once in the composite.

Differences in demographics were stratified by education level and categorical variables were analyzed with Chi-squared tests or Fisher’s tests where appropriate. Rates of adverse outcomes were reported per 1,000 live births. Multivariable modified Poisson regression models with robust error variance were used to detect the differences in the rates of maternal and neonatal morbidity in pregnancies conceived using MAR amongst mothers with differing education levels.

The results were presented as adjusted relative risk (aRR) with a 95% CI. Confounders that were adjusted for included maternal age (< 35, ≥ 35 years), maternal race and ethnicity (non-Hispanic white, non-Hispanic black, Hispanic, non-Hispanic other, unknown), marital status (married, not married), smoking during pregnancy (yes, no, unknown), infant sex (male, female), and delivery year (2016, 2017, 2018, 2019, 2020).

The statistical analysis was conducted using SAS 9.4. (SAS Institute, Cary NC). This study was considered exempt by the institutional review board at the Warren Alpert School of Medicine at Brown University due to the publicly available nature of the dataset, which does not contain direct personal identifiers (IRB# 1746034-1). The STROBE guidelines for reporting observational studies were followed.

## Results

**Fig. 1:**
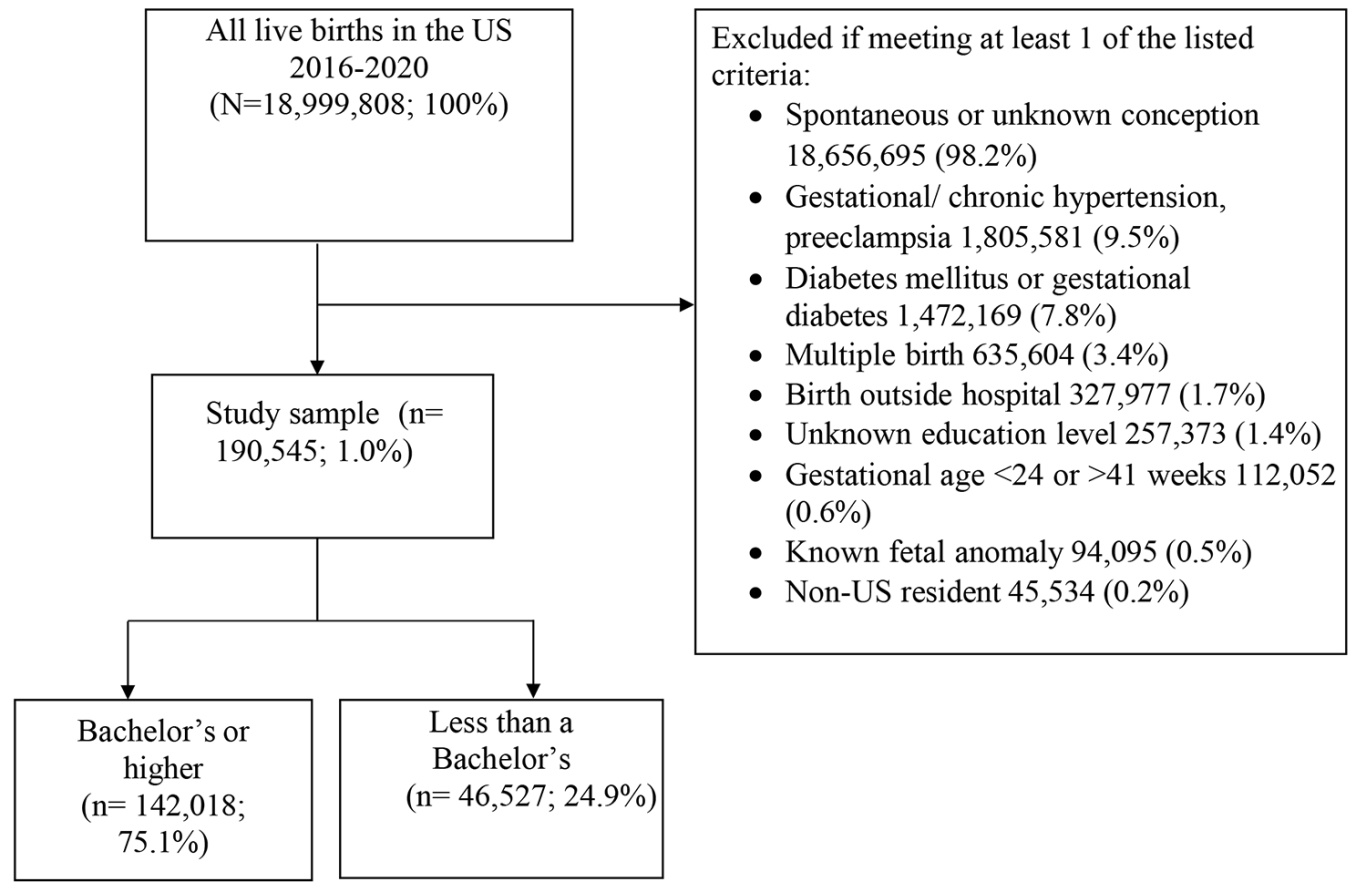
Flow diagram of study population

There were 18,999,808 live births in the study period, of which 190,444 (1.0%) met the inclusion criteria of the study (Fig. 1). The most common reasons for exclusion included: spontaneous or unknown conception method (98.2%), hypertensive disorders (9.5%), and diabetes (7.8%). Most individuals excluded in the spontaneous or unknown conception method group were excluded for spontaneous conception; less than 1% of individuals had a conception method that was unknown.

Demographics are available in Table 1. Maternal demographic data was significantly different between cohorts (p < .001) for all maternal variables. Women with a bachelor’s degree or higher were more likely to be older, non-Hispanic white, clinically normal weight, married, and have private insurance.

**Table 1:** Demographic characteristics of the study population

Characteristic	Total (n = 190,545)	With Bachelor’s degree (n = 143,018, 75.1%)	Without Bachelor’s degree (WBD) (n = 47,527, 24.9%)	P
Maternal age (y)				
Mean (SD)	34.5 (5.1)	35.2 (4.7)	32.4 (5.6)	
< 20	96 (0.1)	0 (0.0)	96 (0.2)	< 0.001
20–34	97,874 (51.4)	66,789 (46.7)	31,085 (65.4)	
35 years or more	92,575 (48.6)	76,229 (53.3)	16,346 (34.4)	
Maternal Race and Ethnicity				
Non-Hispanic white	140,375 (73.7)	107,436 (75.1)	32,939 (69.3)	< 0.001
Non-Hispanic black	8,289 (4.4)	5,267 (3.7)	3,022 (6.4)	
Hispanic	16,166 (8.5)	8,682 (6.1)	7,484 (15.7)	
Non-Hispanic Other	24,539 (12.9)	20,734 (14.5)	3,805 (8.0)	
Unknown	1,176 (0.6)	899 (0.6)	277 (0.6)	
Married				
No	12,962 (6.8)	7,165 (5.0)	5,797 (12.2)	< 0.001
Yes	161,882 (85.0)	123,394 (86.3)	38,488 (81.0)	
Unknown	15,701 (8.2)	12,459 (8.7)	3,242 (6.8)	
Insurance				
Medicaid	9,608 (5.0)	2,896 (2.0)	6,712 (14.1)	< 0.001
Private	173,223 (90.9)	135,369 (94.7)	37,854 (79.6)	
Self-pay	2,449 (1.3)	1,605 (1.1)	844 (1.8)	
Other	4,607 (2.4)	2,747 (1.9)	1,860 (3.9)	
Unknown	658 (0.3)	401 (0.3)	257 (0.5)	
Prenatal Care				
No	395 (0.2)	256 (0.2)	139 (0.3)	< 0.001
Yes	187,065 (98.2)	140,453 (98.2)	46,612 (98.1)	
Unknown	3,085 (1.6)	2,309 (1.6)	776 (1.6)	
Smoking during pregnancy				
No	189,045 (99.2)	142,566 (99.7)	46,479 (97.8)	< 0.001
Yes	1,168 (0.6)	238 (0.2)	930 (2.0)	
Unknown Pre-pregnancy BMI (kg/m^2^)	332 (0.2)	214 (0.1)	118 (0.2)	
Mean (SD)	26.7 (10.2)	26.1 (10.1)	28.6 (10.5)	
Underweight (< 18.5)	4,605 (2.4)	3,665 (2.6)	940 (2.0)	< 0.001
Normal weight (18.5–24.9)	98,989 (52.0)	80,701 (56.4)	18,288 (38.5)	
Overweight (25.0-29.9)	47,545 (25.0)	34,349 (24.0)	13,196 (27.8)	
Obesity I (30.0-34.9)	21,672 (11.4)	13,865 (9.7)	7,807 (16.4)	
Obesity II (35.0-39.9)	9,835 (5.2)	5,746 (4.0)	4,089 (8.6)	
Extreme Obesity III (≥ 40.0)	5,383 (2.8)	2,794 (2.0)	2,589 (5.4)	
Unknown	2,516 (1.3)	1,898 (1.3)	618 (1.3)	
Prior preterm delivery				
No	185,139 (97.2)	139,412 (97.5)	45,727 (96.2)	< 0.001
Yes Gestational Age at Delivery	5,406 (2.8)	3,606 (2.5)	1,800 (3.8)	
Mean (SD)	38.7 (1.9)	38.7 (1.9)	38.5 (2.1)	
24–36 weeks	15,748 (8.3)	11,254 (7.9)	4,494 (9.5)	
37–41 weeks	174,797 (91.7)	131,764 (92.1)	43,033 (90.5)	
Infant sex				
Female	93,647 (49.1)	70,569 (49.3)	23,078 (48.6)	0.003
Male	96,898 (50.9)	72,449 (50.7)	24,449 (51.4)	
Route of delivery				
Cesarean	80,649 (42.3)	60,890 (42.6)	19,759 (41.6)	< 0.001
Operative vaginal	10,183 (5.3)	7,952 (5.6)	2,231 (4.7)	
Spontaneous vaginal	99,671 (52.3)	74,145 (51.8)	25,526 (53.7)	
Unknown	42 (0.0)	31 (0.0)	11 (0.0)	
Delivery year				
2016	33,051 (17.3)	24,343 (17.0)	8,708 (18.3)	< 0.001
2017	36,607 (19.2)	27,421 (19.2)	9,186 (19.3)	
2018	38,713 (20.3)	29,039 (20.3)	9,674 (20.4)	
2019	41,278 (21.7)	31,081 (21.7)	10,197 (21.5)	
2020	40,896 (21.5)	31,134 (21.8)	9,762 (20.5)	

Abbreviation: MAR, Medically assisted reproduction; WBD, without bachelor’s degree

Notes: Data presented as n (%)

The overall rate of maternal morbidity amongst individuals included in the sample was 9.9 per 1,000 live births. Composite maternal adverse outcomes were similar among patients with a bachelor’s degree (10.1 per 1,000 live births) and those without a bachelor’s degree (9.4 per 1,000 live births); ARR 1.05, 95% CI (0.94–1.17) (Table 2). There was no difference between groups of any subcomponents of the composite outcome.

**Table 2:** Composite and Individual Maternal Morbidity for individuals with and without a Bachelor’s Degree who conceived using Medically Assisted Reproduction

Outcome		Total Live Births	n	Rate/1,000 Live Births	Adjusted RR (95% CI)
Composite maternal morbidity	Total	190,444	1,893	9.9	
	Bachelors	142,943	1,448	10.1	1.05* (0.94–1.17)
	WBD	47,501	445	9.4	Ref
Admission to ICU	Total	190,444	435	2.3	
	Bachelor’s	142,943	347	2.4	1.21^†^ (0.94–1.54)
	WBD	47,501	88	1.9	Ref
Maternal transfusion	Total	190,444	1,554	8.2	
	Bachelor’s	142,943	1,191	8.3	1.06* (0.94–1.19)
	WBD	47,501	363	7.6	Ref
Uterine rupture	Total	190,444	91	0.5	
	Bachelor’s	142,943	69	0.5	1.02^†^ (0.63–1.64)
	WBD	47,501	22	0.5	Ref
Unplanned hysterectomy	Total	190,444	230	1.2	
	Bachelor’s	142,943	183	1.3	1.16^†^ (0.82–1.63)
	WBD	47,501	47	1.0	Ref

RR, relative risk; CI, confidence interval; ICU, intensive care unit

Notes: 101 individuals were excluded due to missing information on maternal morbidity

*Adjusted for maternal age (< 35 vs. 35+), race and ethnicity, marital status, prenatal care, smoking during pregnancy, neonatal sex, and birth year

^†^Adjusted for maternal age (< 35 vs. 35+), race and ethnicity, marital status, neonatal sex, and birth year

The overall rate of neonatal morbidity amongst individuals included in the sample was 97.0 per 1,000 live births. Composite adverse neonatal outcomes were significantly lower in women with a bachelor’s degree or higher (94.1 per 1,000 live births) compared to women without a bachelor’s degree (105.9 per 1,000 live births); ARR 0.91, 95% CI (0.88–0.94) (Table 3). Rates of NICU admission and ventilator support differed between cohorts.

**Table 3:** Composite and Individual Neonatal Morbidity for individuals with and without a Bachelor’s Degree who conceived using Medically Assisted Reproduction

Outcome		Total Live Births	n	Rate/1,000 Live Births	Adjusted RR (95% CI)
Composite neonatal morbidity	Total	190,517	18,480	97.0	
	Bachelor’s	143,998	13,449	94.1	**0.91** **(0.88–0.94)**
	WBD	47,519	5,031	105.9	Ref
Admission to NICU	Total	190,517	18,204	95.6	
	Bachelor’s	143,998	13,247	92.6	**0.91** **(0.88–0.94)**
	WBD	47,519	4,957	104.3	Ref
Ventilator support	Total	190,517	3,926	20.6	
	Bachelor’s	143,998	2,718	19.0	**0.77** **(0.72–0.83)**
	WBD	47,519	1,208	25.4	Ref
Seizure	Total	190,517	115	0.6	
	Bachelor’s	143,998	91	0.6	1.32 (0.83–2.09)
	WBD	47,519	24	0.5	Ref

RR, relative risk; CI, confidence interval; ICU, intensive care unit; WBD, without bachelor’s degree; IUI, intrauterine insemination; IFM, infertility medications

Notes: 28 individuals were excluded due to missing information on neonatal morbidity

Adjusted for maternal age (< 35 vs. 35+), race and ethnicity, marital status, prenatal care, smoking during pregnancy, neonatal sex, and birth year

The preplanned sub analysis demonstrated that in both cohorts women receiving intrauterine insemination or infertility medications experienced less maternal morbidity than women undergoing in vitro fertilization (Table 4). In women with a bachelor’s degree the difference was 6.5 vs. 11.9 per 1,000 live births; ARR 0.56, 95% CI (0.49–0.63). In women without a bachelor’s degree, it was 6.7 vs. 11.6 per 1,000 live births; ARR 0.58, 95%CI (0.47–0.71).

**Table 4:** Composite Maternal Morbidity by Infertility Treatment Stratified by Education Level

Outcome		Total Live Births	n	Rate/1,000 Live Births	Adjusted RR (95% CI)
Bachelors+	Total	142,943	1,448	10.1	
	IUI/IFM	47,457	308	6.5	**0.56** **(0.49–0.63)**
	IVF	95,486	1,140	11.9	Ref
Without college degree	Total	47,501	445	9.4	
	IUI/IFM	21,288	142	6.7	**0.58** **(0.47–0.71)**
	IVF	26,213	303	11.6	Ref

RR, relative risk; CI, confidence interval; ICU, intensive care unit; IUI, intrauterine insemination; IFM, infertility medications

Notes: 101 individuals were excluded due to missing information on maternal morbidity Adjusted for maternal age (< 35 vs. 35+), race and ethnicity, marital status, neonatal sex, and birth year

## Discussion

This population-based retrospective cohort study demonstrated no significant association between lower levels of maternal education and increased maternal morbidity in individuals who conceived using MAR, even after adjustment for potential cofounders. There was, however, an association between maternal education and neonatal morbidity. There have been several studies that describe the relationship between formal education and health with education being cited as a critical component of a person’s health and a factor in other elements of a person’s current and future health. People with higher education levels often have access to health promoting resources while people with lower education have less access to these resources and often have unhealthy work environments [17, 18].

There is a growing body of literature identifying maternal race and ethnicity as independent risk factors for poor reproductive and neonatal outcomes in both spontaneous pregnancies and in pregnancies achieved using MAR [19–21]. More recently, researchers have pivoted from focusing solely on race to exploring the other, potential targetable, socioeconomic determinants of health potentially contributing to the growing disparities in reproductive outcomes of people of color [11]. Increased education level and health literacy, for example, have been associated with improved health outcomes [11, 22, 23]. However, few studies assessing the role of maternal education in maternal and neonatal morbidity exist due to missing data on maternal education status from many large databases; in these situations, race is often used as representation for socioeconomic status, leaving the effect of maternal education on morbidity largely unknown. In one study, lower maternal education was associated with a higher incidence of maternal and neonatal morbidity compared to women with a 4-year college degree, though this study did not differentiate between spontaneously achieved pregnancies and pregnancies achieved using MAR [11].

State mandated coverage of IVF and assisted reproductive technology has allowed for more inclusive access to infertility treatment, particularly in patient populations with lower income and lower health literacy. However, despite improvements in access to infertility care, disparities in outcomes still exist. In fact, racial disparities in utilization not only persisted regardless of mandate, but were greater in mandated states [24]. Our findings suggesting that there is no significant association between lower levels of maternal education and increased maternal morbidity in individuals who conceived using MAR initially appears to be inconsistent with trends in disparities found in other studies. However, it suggests that further research should be done to investigate other socioeconomic determinants of health as potential contributors to the disparities in outcomes that exist when using MAR.

There are several strengths to this study. The sample size of over 190,000 individuals provides power to detect uncommon outcomes such as unplanned hysterectomy and neonatal seizures. The use of data from 2016 to 2020 provides a contemporary estimate of rates of maternal and neonatal morbidity in individuals using MAR, and the importance of National Vital Statistics data has been recognized by the American College of Obstetricians and Gynecologists. Limitations of the study include the individuals who were excluded from the study due to unknown method of conception from 2016 to 2020. It is unknown if some of these excluded individuals conceived using MAR and had maternal and neonatal outcomes that could have impacted our findings. Another limitation of this study are the outcomes included in maternal morbidity. Further outcome data was not collected by the national dataset and therefore precludes deeper analysis. Finally, excluding the second parent’s education status also is a limitation of this study. Previous studies have shown strong relationships between paternal education and infant health outcomes, though there are few studies examining the relationship between paternal education and maternal and neonatal outcomes [25].

## Conclusion

This study demonstrated that lower maternal education level was not associated with maternal adverse outcomes in patients who conceived using medically assisted reproduction but was associated with increased rates of neonatal adverse outcomes. As access to infertility care increases, patients who conceive with medically assisted reproduction may be counseled that education level is not associated with maternal morbidity. Further research into the association between maternal education level and neonatal morbidity is indicated.

## Data Availability

Data will be made available to the editors of the journal for review or query upon request.

## Abbreviations

MAR: Medically assisted reproduction
ICU: Intensive Care Unit
RR: Relative Risk
CI: Confidence Interval
CDC: Centers for Disease Control and Prevention
